# Editorial: Obesity and Diabetes: Energy Regulation by Free Fatty Acid Receptors

**DOI:** 10.3389/fendo.2015.00178

**Published:** 2015-11-20

**Authors:** Atsuhiko Ichimura, Ikuo Kimura

**Affiliations:** ^1^Department of Biological Chemistry, Graduate School of Pharmaceutical Science, Kyoto University, Kyoto, Japan; ^2^Center for the Promotion of Interdisciplinary Education and Research, Kyoto University, Kyoto, Japan; ^3^Tokyo University of Agriculture and Technology, Tokyo, Japan, Kyoto, Japan

**Keywords:** obesity, diabetes, energy regulation, free fatty acid receptors, editorial

Food intake regulates energy balance, and its dysregulation leads to metabolic disorders, such as obesity and type 2 diabetes (T2D). During feeding, free fatty acids (FFAs) are not only essential nutrients but also act as signaling molecules in various cellular processes. Recently, several G protein-coupled receptors (GPCRs) that act as FFA receptors (FFARs) have been identified; GPR41/FFAR3 and GPR43/FFAR2 are activated by short-chain FFAs. GPR40/FFAR1, GPR119, and GPR120/FFAR4 are activated by medium- and long-chain FFAs. FFARs are widely expressed and contribute to many important physiological functions in order to maintain energy homeostasis. Hence, these FFARs have come to be regarded as new drug targets for metabolic disorder such as obesity and T2D.

All articles in this topic highlight the interconnection between FFARs and the regulation of energy homeostasis. They also focused on essential role of FFARs in the pathogenesis of metabolic syndromes, such as obesity, insulin resistance, and T2D and discussed the potential of FFARs as drug target. These articles give valuable insight into unanswered questions in relation to this topic. First, recent studies demonstrate that short-chain free fatty acids (SCFAs) produced by microbiota fermentation act as signaling molecules through SCFAs receptors (SCFARs), such as GPR41 and GPR43 and influence the host’s metabolism (1–3). Hence, the gut microbiota can influence and play important roles in host physiology and pathology *via* these receptors. GPR41, which is expressed in adipose tissue, gut, and the peripheral nervous system, contributes SCFAs-dependent systemic energy regulation (1). In particular, GPR41 regulates host energy balance by modulating sympathetic activity and intestinal gluconeogenesis. GPR43, which is expressed in the adipose tissue, intestines, and immune tissues, also contributes the regulation of energy homeostasis depends on SCFAs produced by gut microbiota (2). GPR43 deficiency induced obesity in mice, while mice that overexpress GPR43 only in adipose tissue were lean under normal conditions; both of these strains did not exhibit either phenotype under germ-free conditions or after antibiotic treatment. Furthermore, SCFA-mediated GPR43 activation suppressed adipose insulin signaling, leading to inhibition of fat accumulation in the adipose tissues, while unincorporated lipids and glucose were primarily utilized in muscles. The GPR43-insulin pathway has a key role in adipose tissue acting as an important physiological mechanism through which metabolic fuels regulate body energy balance (2, 3). These studies clearly showed the importance of SCFAs produced by microbiota and their receptors (1–3). Based on the importance and dynamic roles of microbiota in host physiology, Pluznick pointed out a complex interplay between the genetics of the microbiota and that of the host organism (4). Researchers should consider the contribution of these microorganisms and their metabolites because there are many examples of phenotypes that were not easily to replicated by other groups may be due to the influence of variations of gut microbiota (4). Second, medium-chain fatty acids (MCFAs) and long-chain fatty acids (LCFAs) are not only essential nutrient, but also act as ligands of GPR40/FFAR1 and GPR120/FFAR4 and regulate systemic energy homeostasis (5–8). GPR40 is highly expressed in pancreatic β cells and intestine. GPR40 augment glucose-stimulated insulin secretion after acute exposure to LCFAs by stimulation of not only insulin secretion from pancreatic β cells directly, but also incretin hormones, such as glucagon like peptide-1 (GLP-1), gastric inhibitory polypeptide (GIP) and cholecystokinin (CCK) from intestine (5, 8). The activation of GPR120 by ω-3 FFAs mediated anti-inflammatory effect of ω-3 FFAs as described in the articles by Oh et al. (7). This effect is associated with the suppression of Toll-like receptor via β-arrestin2 signaling pathway and transforming growth factor-β activated kinase 1 (TAK1) involved in TNF-α inflammation signaling pathway. Furthermore, both a gene deficiency in mice and non-synonymous functional-loss mutation of human GPR120 are associated with obesity, which was accompanied with decreased differentiation and lipogenesis (6). Hence, selective synthetic ligands for FFARs have consequently been developed as potential treatments for metabolic syndrome (9). Particularly, clinical studies show that TAK875/Fasiglifam, an agonist of GPR40 improved glucose metabolism with a reduced risk of hypoglycemia, although this ligand was dropped from clinical trials due to potential liver toxicity. Activation of each of GPR41, 43, and 120 has also been suggested to have potential benefits for metabolic function (9).

Overall, all the review articles provided a comprehensive overview of the energy regulation by FFARs and a new prospect for treatment of metabolic disorder such as obesity and type 2 diabetes.

## Conflict of Interest Statement

The authors declare that the research was conducted in the absence of any commercial or financial relationships that could be construed as a potential conflict of interest.
